# Practical issues and limitations of brain attenuation correction on a simultaneous PET-MR scanner

**DOI:** 10.1186/s40658-020-00295-x

**Published:** 2020-05-05

**Authors:** J. E. Mackewn, J. Stirling, S. Jeljeli, S-M. Gould, R. I. Johnstone, I. Merida, L. C. Pike, C. J. McGinnity, K. Beck, O. Howes, A. Hammers, P. K. Marsden

**Affiliations:** 1 King’s College London and Guy’s and St Thomas’ PET Centre, School of Biomedical Engineering and Imaging Sciences, King’s College London, London, UK; 2 Guy’s and St. Thomas’ NHS Foundation Trust, London, UK; 3 CERMEP-Imagerie du vivant, Lyon, France; 4 Department of Psychosis Studies, Institute of Psychiatry, Psychology and Neuroscience, King’s College London, De Crespigny Park, London, UK; 5 South London and the Maudsley NHS Foundation Trust, London, UK; 6 MRC London Institute of Medical Sciences, Hammersmith Hospital Campus, London, UK

**Keywords:** PET-MR, Brain, Linear attenuation coefficient, PET-CT, Padding, Hair, Headphones

## Abstract

**Background:**

Despite the advent of clinical PET-MR imaging for routine use in 2011 and the development of several methods to address the problem of attenuation correction, some challenges remain. We have identified and investigated several issues that might affect the reliability and accuracy of current attenuation correction methods when these are implemented for clinical and research studies of the brain. These are (1) the accuracy of converting CT Hounsfield units, obtained from an independently acquired CT scan, to 511 keV linear attenuation coefficients; (2) the effect of padding used in the MR head coil; (3) the presence of close-packed hair; (4) the effect of headphones. For each of these, we have examined the effect on reconstructed PET images and evaluated practical mitigating measures.

**Results:**

Our major findings were (1) for both Siemens and GE PET-MR systems, CT data from either a Siemens or a GE PET-CT scanner may be used, provided the conversion to 511 keV μ-map is performed by the PET-MR vendor’s own method, as implemented on their PET-CT scanner; (2) the effect of the head coil pads is minimal; (3) the effect of dense hair in the field of view is marked (> 10% error in reconstructed PET images); and (4) using headphones and not including them in the attenuation map causes significant errors in reconstructed PET images, but the risk of scanning without them may be acceptable following sound level measurements.

**Conclusions:**

It is important that the limitations of attenuation correction in PET-MR are considered when designing research and clinical PET-MR protocols in order to enable accurate quantification of brain PET scans. Whilst the effect of pads is not significant, dense hair, the use of headphones and the use of an independently acquired CT-scan can all lead to non-negligible effects on PET quantification. Although seemingly trivial, these effects add complications to setting up protocols for clinical and research PET-MR studies that do not occur with PET-CT. In the absence of more sophisticated PET-MR brain attenuation correction, the effect of all of the issues above can be minimised if the pragmatic approaches presented in this work are followed.

## Background

For a PET image to be quantifiable, and thus represent an accurate distribution of activity, there are a series of data corrections that are needed (e.g. for attenuation, scatter and randoms). The most important of these corrections in PET is attenuation correction (AC), which affects both the visual quality and the quantitative accuracy of a PET image. In PET-CT the necessary 511 keV attenuation correction factors can be derived directly from a CT scan, provided the difference in the attenuating properties of materials at low CT X-ray energies and PET 511 keV gamma ray energies is accounted for, as well as the difference in spatial resolution between a CT and a PET image. MR images, however, reflect a combination of tissue relaxation properties and proton density, which are not simply related to 511 keV gamma-ray linear attenuation coefficients. A wide variety of methods have been proposed and evaluated for attenuation correction of PET data in PET-MR imaging. Current techniques that have been fairly widely used for brain attenuation correction are as follows:

1. Using a CT scan measured independently on a PET-CT scanner. This requires the development of a process to import a registered CT-derived μ-map into the PET-MR reconstruction pipeline.
2. Using a segmentation algorithm applied to an MR image acquired on the PET-MR scanner that assigns different tissue types to a small number of different tissue classes (e.g. soft tissue, bone, air). Attenuation factors are then applied to each class to generate a μ-map. To account for bone, a bone atlas can be directly incorporated into the MR-derived μ-map or the underlying MR sequence has a very short echo time (e.g. ultra-short echo time (UTE) or zero echo time (ZTE)) to generate signal from bone that can be included as an additional tissue class. Algorithms to do this are available on current PET-MR systems [1–4].
3. Atlas-based approaches that estimate a patient-specific μ-map by performing registration of patient’s anatomic MR images to a CT based atlas derived from a database of CT images [5] or a dataset of paired MR and CT images [6, 7]. Currently, only the method described by Wollenweber et al. [5] is implemented on a commercial PET-MR scanner.

A review of these methods, and other techniques, can be found in work by Mehranian et al. [8], with additional examples given by Lui et al. [9] and García-Pérez et al. [10].

For neuroimaging, the problem of attenuation correction in PET-MR for adults with normal anatomy is widely considered solved. This was also concluded in the report by Bailey et al. [11]. Furthermore, in a multicentre study that compared 11 different methods, for the best performing techniques only very small differences (e.g. ± 3%) were recorded when compared to using CT for attenuation correction as reported by Ladefoged et al. [12]. Similar small errors were also reported in a more recent study to evaluate template-enhanced zero-echo-time attenuation correction by Delso et al. [4]. However, although the best of these methods can produce an accurate representation of an ideal CT-derived μ-map inside the boundary of the patient’s head, there are still a number of outstanding practical issues that are not addressed when faced with the task of how to reliably derive a 511 keV μ-map for routine processing of large patient cohorts in quantitative PET-MR studies or even routine clinical scans. Here we describe and address several of these issues that can hinder the robust practical implementation of PET-MR attenuation correction in neuroimaging.

### Converting Hounsfield units (HUs) to 511 keV linear attenuation coefficients (LACs) for attenuation correction using a separately acquired CT scan

**Fig. 1:**
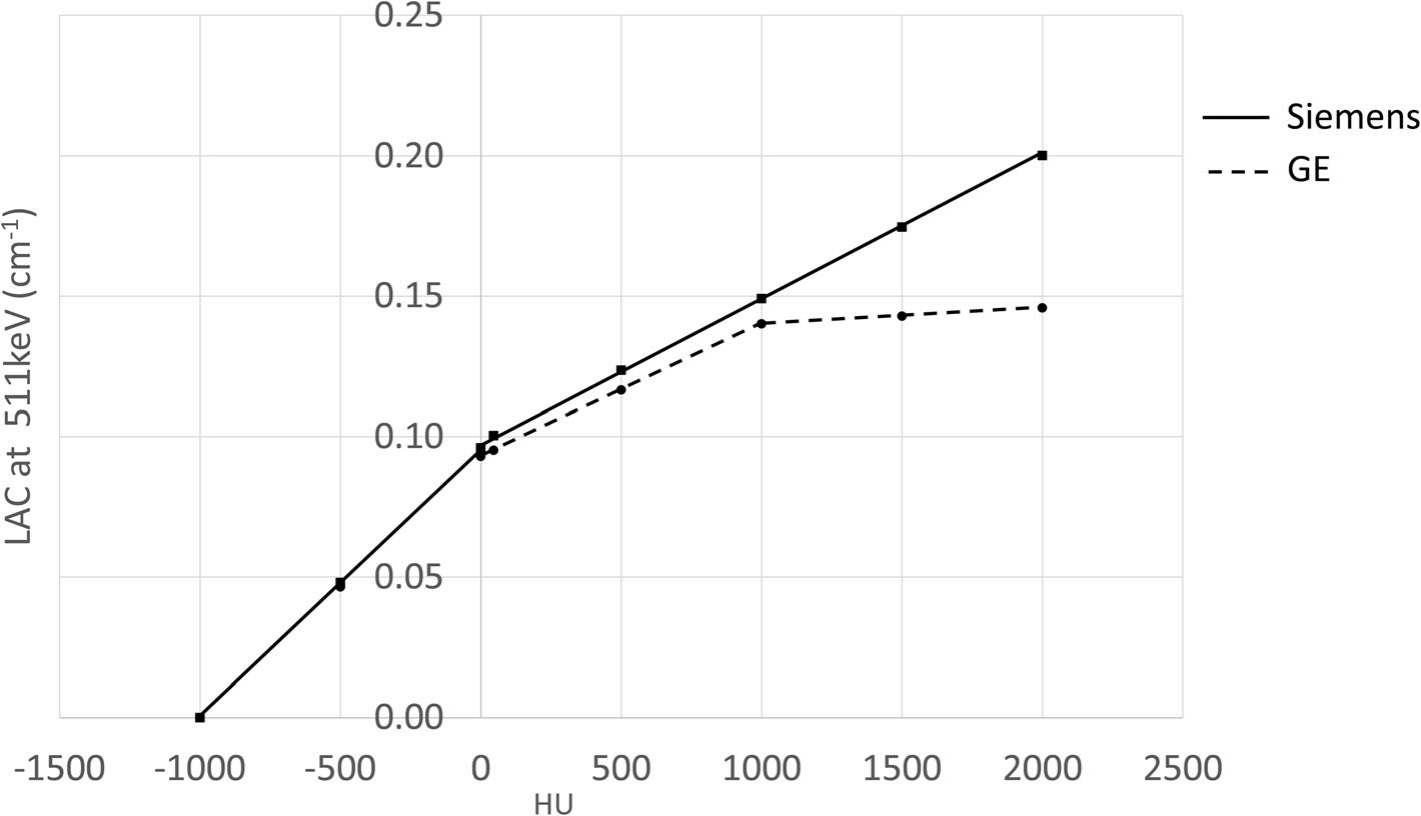
Curves used by different vendors to scale Hounsfield unit in a 120 kVp CT image to LAC at 511 keV

If a CT image, e.g. acquired on a PET-CT scanner, is used for attenuation correction of the PET data acquired on a PET-MR scanner, scaling must be applied to convert the HUs of the CT image to LAC at 511 keV. Different vendors of PET-CT scanners use different transformations for this conversion. The graph in Fig. 1 shows the trilinear approximation used by GE [13–15] and the bilinear approximation used by Siemens [16] on their PET-CT scanners, both in the absence of contrast agent and for a tube voltage of 120 kVp. It is not clear which curve should be used if the PET-CT and PET-MR are from different vendors, as may often be the case in practice.

### Effect of pads used to position and keep the patient’s head still during long PET-MR examinations

Pads are often required to keep the patient’s head stationary during the scan. This is of particular importance in research brain imaging as the scan duration is generally long (e.g. 1–2 h) in order to acquire a large number of MR sequences and/or to accommodate slow PET tracer kinetics. These pads are not necessarily included in the MR-derived μ-map due to uncertainty in their position during a scan. Their presence could potentially result in a heterogeneous decrease in activity concentration and artefacts in reconstructed PET images.

### Effect of dense hair in the FOV

**Fig. 2:**
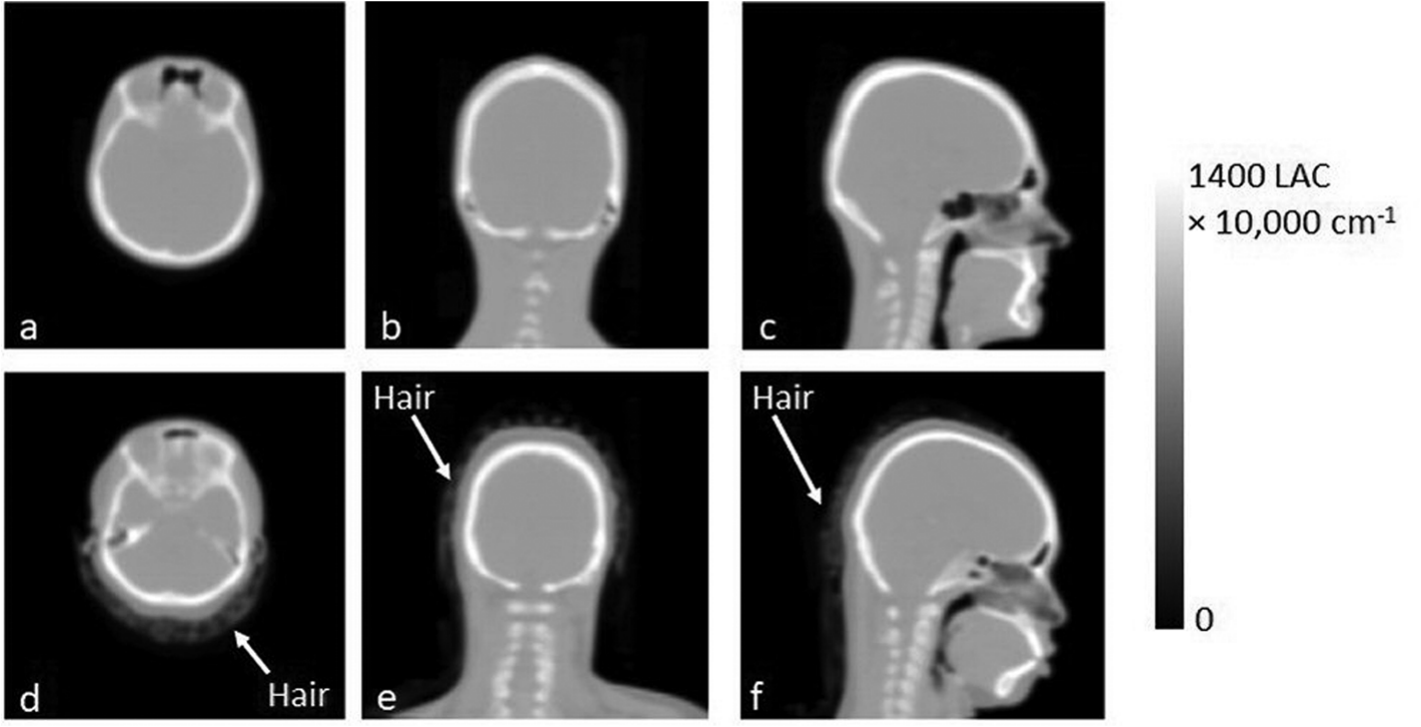
**a**–**c** CT-derived μ-map of a patient who has short/low density hair. **d**–**f** CT-derived μ-map of a patient with dense hair surrounding the head (arrows indicate location of the hair in each image)

In neurological and psychiatric studies undertaken on our PET-MR scanner, we have encountered a number of subjects who had dense/clumped hair, e.g. those with braids, dreadlocks and pony tails. These correspond to reasonably high HU values (e.g. − 500 to − 300) on CT. Hair is immediately adjacent to the scalp. It is not incorporated as a different tissue class in the segmented MRI-derived μ-maps available on the scanners that use ZTE, UTE or a Dixon sequence. If present, the small heterogenous signal from hair gets assigned to air. Hair is also not accounted for in atlas based methods. The unaccounted-for attenuation by the hair in such μ-maps can result in errors in the reconstructed PET images. Fig. 2a–c shows a CT-derived μ-map of a subject who appears to have only short/low density hair. There is little signal surrounding the patient’s scalp, compared to a CT-derived μ-map of a different subject’s head in which hair can be seen as far down as the base of the neck (Fig. 2d–f).

### Effect of headphones and safety considerations when they are not worn

On a 3T scanner (the field strength used in current commercial PET-MR scanners), it can be recommended that headphones are used in addition to ear plugs. These headphones, although made from plastic, are quite thick (~ 2 cm wide with foam padding on each side) and will attenuate 511 keV gamma rays, but are not easily included in an MR-derived μ-map. Siemens advise that they should not be used when acquiring PET images on the mMR as, unlike the head coil, they are not accounted for in the attenuation map. The effect of headphones has been reported previously [17–20]. Our work complements these reports by providing an assessment using a phantom with a realistic geometry on the Siemens Biograph mMR. In addition we also provide measurements of sound levels that demonstrate under most circumstances it is safe to scan without headphones, thus eliminating the problem altogether.

## Materials and methods

The work presented here is primarily focussed around attenuation correction for PET images acquired on the Siemens Biograph mMR PET-MR scanner [21]. The mMR is the first generation of commercial simultaneous whole body PET-MR scanners. It has a 3T magnet, uses avalanche photodiodes (APDs) for the PET photodetectors and lacks time of flight (TOF) capability due to limitations in the timing performance of the APDs. Attenuation correction provided on this scanner for the brain is either using a segmented UTE μ-map [2] or a Dixon sequence that incorporates a bone Atlas [1]. The head coil and bed are included in the attenuation map at their known physical location. Most of our results are expected to also apply to other PET-MR scanners (at least under similar imaging conditions, i.e. without TOF) in particular to the more recent GE SIGNA scanner, which incorporates silicon photomultipliers as the PET photo detectors [22].

For the patient images used as part of this evaluation full ethics approval was in place with signed consent from each participant for use of their images (details of the ethics committees are given in the declaration sections below). The patient images are taken from a study to investigate psychosis using PET-MR with a glutamate receptor binding agent. However the results are generally applicable to other patient groups and tracers, particularly as the methods of attenuation correction used throughout are independent of the emission data.

We use three different phantoms in the assessments described below. Where possible, the ^68^Ge uniform daily QC cylinder (diameter = 20 cm, length = 40 cm) is used as it is straightforward to image using a CT-derived μ-map available on the PET-MR scanner. For the comparison of CT images acquired on Siemens and GE systems, we used an electron density phantom (Virtual Water™ supplied by TomoTherapy Inc. [23]) to provide a range of different density materials. For the assessment of the headphones we used a realistic head shaped phantom with a uniform region the size of a brain surrounded by skull [24]. Other groups [17–20] have performed this assessment with the ^68^Ge cylinder however these give an unduly pessimistic result as the headphones are located in very close proximity to the activity of the phantom. This does not occur in the brain due to the presence of the skull.

Unless otherwise stated, all PET and CT images were acquired and processed using a local brain acquisition and reconstruction method. The PET scans are reconstructed on the mMR scanner with randoms and scatter correction applied using ordered subsets expectation maximisation (OSEM) with 2 iterations and 21 subsets, a 256 × 256 × 127 matrix and a 4-mm post reconstruction smoothing filter. The method of attenuation correction is described within each section below. The CT is acquired on a GE Discovery 710 in helical mode at 140 kVp or 120 kVp (for adults and paediatrics respectively), 8 mAs with a pitch of 1.375, slice thickness of 3.27 mm and reconstructed using GE’s standard filtered back projection (FBP) method. The research brain scans, described below, were reconstructed using a PET frame from 65 to 75 min after the injection of the ^18^F-based glutamate receptor binding agent.

### Converting HUs to 511 keV LACs for attenuation correction using a separately acquired CT scan

To investigate how best to scale CT images from a PET-CT scanner for attenuation correction of PET data from PET-MR, we measured the difference between HUs and LACs derived from images acquired on GE and Siemens PET-CT systems. The CT electron density phantom was scanned using the standard brain protocol on a Siemens Biograph mCT and a GE Discovery 710 PET-CT scanner at 120 kVp. The Siemens brain CT protocol is acquired in helical mode at 8 mAs, with a pitch of 1.35 and slice thickness of 3 mm. The images were reconstructed with FBP using Siemens standard soft tissue filter. The phantom consists of a cylinder made from ‘solid water’ (an epoxy resin material designed to mimic the attenuating properties of water) into which cylindrical samples of known density are inserted. The samples have a diameter of approximately 25 mm. ROI values for the six most dense samples (densities ranging from 1.051 g cm^−3^ (brain) to 1.882 g cm^−3^ (bone) covering the range of tissue densities seen in the head), were compared both in HU and after conversion to 511 keV LAC.

To investigate the effect of the choice of scaling curve, on reconstructed PET activity concentration, the same PET scan of the daily ^68^Ge quality control uniform cylinder (acquired using a 5-min frame on the mMR) was attenuation corrected using (i) the built-in Siemens CT-derived μ-map available on the mMR for routine reconstruction of the phantom, (ii) a separately acquired GE CT scan with Siemens scaling applied and (iii) a separately acquired GE CT scan with GE scaling applied. The activity concentrations measured in the resulting attenuation corrected PET images were compared.

### Effect of pads used to position and keep the patient’s head still during long brain PET-MR examinations

**Fig. 3:**
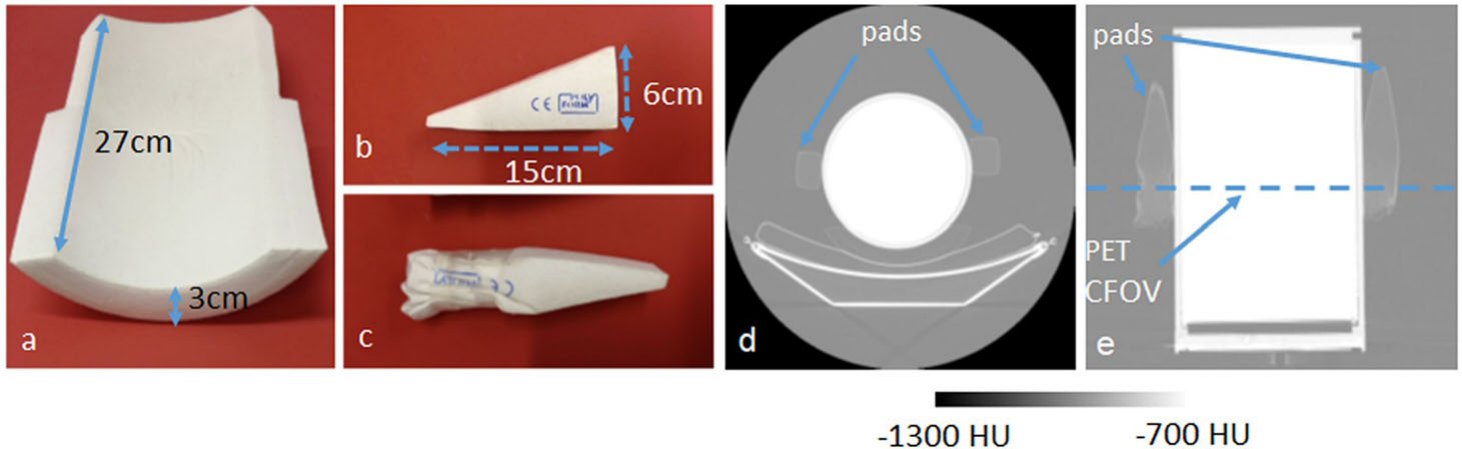
**a** Pad located under the patient’s head in the head coil. **b** A triangular pad (used in pairs) that is positioned between the coil and the head. **c** The same triangular pad compressed with tape to mimic compression when in use. **d** Transverse and **e** coronal CT images in HU showing the location of the pads for phantom assessment (CFOV = centre of the field of view). The blue arrows in **d** and **e** highlight the location of the pads in the CT images

To assess the effects of the three pads commonly used in the head coil of the mMR, the pads were attached to the cylindrical daily QC phantom. The phantom contained approximately 100 MBq of ^68^Ge. The pads are shown in Fig.3a–c, along with a CT image of the phantom with the pads attached in Fig. 3d, e. As seen in the CT in Fig. 3e, both triangular pads were compressed with tape (as shown in Fig. 3c) in order to mimic compression between the head coil and the patients head when in use. The pads are made from a low-density polyurethane foam in a polyvinyl chloride (PVC) cover. The CT number of the cushion underneath the phantom was approximately − 950 HU. In the side pads, the HU increased from − 900 HU to − 850 HU in the area compressed with tape. The phantom was suspended off the end of the bed using the Siemens phantom holder so that the PET images could be attenuation corrected using the ^68^Ge CT-derived μ-map available on the scanner. The daily QC phantom was chosen for this test as it provides a worst case assessment of the low density pads as they are much closer to the activity in the phantom than they would be to the uptake in the brain. If the pads have a negligible effect in this imaging scenario they are highly unlikely to affect the accuracy of quantification in the brain.

A PET scan of the ^68^Ge phantom was acquired on the mMR scanner for 20 min with the pads in place. Without moving the bed between acquisitions, the pads were removed and a further 20-min scan was acquired. A third acquisition of 20 min was performed without the pads to look at the variability between two PET scans acquired back to back. To quantify the potential error imposed by a small patient shift between the acquisition of the μ-map and a PET scan, i.e. the type of small movement likely to occur in the absence of the pads, an additional image was reconstructed with a 2-mm shift in the transverse plane between PET image and the CT-derived μ-map.

### Effect of dense hair in the FOV

The effect of hair was quantified for the subject shown in Fig. 2d–f who had hair clearly visible around the head. A manual ROI was drawn on each transverse slice of the CT to carefully remove the hair without removing any of the scalp. The PET scan of this subject, acquired on the mMR, was attenuation-corrected using the CT with the hair included and alternatively using the CT with the hair cropped out of the image.

The effect of hair was also indirectly assessed on 14 subjects as part of an evaluation of a state-of-the-art atlas-based MRAC method called MaxProb [6]**,** undertaken at our institution. MaxProb outperformed the UTE and Dixon-based segmentation methods implemented on the mMR and was one of the best performing brain attenuation correction methods reported by Ladefoged et al. [12]. The method generates a pseudo-CT image through the registration of a database of aligned MRI and CT image pairs with a T1-weighted MRI image acquired as part of the PET-MR scanning protocol. Hair is not included in the pseudo-CT image—all pixels located outside the boundary between air and the patient’s scalp are assigned an LAC value of zero. For each subject, an image was generated of the percentage difference between a static PET image attenuation-corrected using the CT-derived μ-map from a GE PET CT scanner and an image reconstructed using a pseudo-CT generated by MaxProb.

### Effect of headphones and safety considerations when they are not worn

To investigate the effect of using headphones, the brain phantom [24], with a geometry comparable to that of a subject’s head, was filled with approximately 50 MBq of ^18^F and scanned on the PET-MR scanner for 20 min with and without the headphones in place. The headphones were supplied by Siemens (model: 10018373, revision 6 11 November 2015). A CT scan of the phantom, without the headphones, was used for attenuation correction. An image was calculated of the difference between the PET images of the phantom scanned with the headphones on and with the headphones off, with a decay correction applied for ^18^F.

To ascertain if harm would be caused to the patient’s hearing if the headphones were not used, sound risk assessments were performed for four brain scans with scan duration’s varying between 1 to 2 h. These studies were comprised of a range of anatomical T1- and T2-weighted images and quantitative techniques such as spectroscopy, arterial spin labelling and resting state functional MRI echo planar imaging (fMRI-EPI). It was found that generally the EPI sequences were the loudest. For each study, the A-weighted average noise level (*L*_Aeq_) and peak noise level (*L*_Zpk_) [25] were recorded, using a microphone located in the scanner, for the duration of all the sequences. The noise levels actually experienced by the patient were then calculated by taking into account the noise reduction rating (NRR) of the 3M Taperfit 2 ear-plugs worn by all patients.

## Results

### Converting HUs to 511keV LACs for attenuation correction using a separately acquired CT scan

**Fig. 4:**
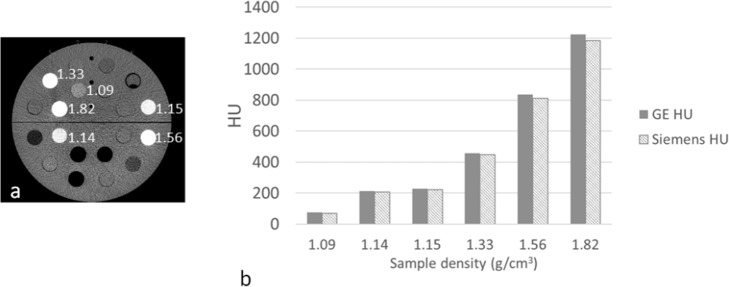
**a** GE CT image of the electron density phantom labelled with the density of each sample. The image is displayed in HU. **b** HU measured on a GE and Siemens CT image of the phantom

The HUs measured on a GE and Siemens scanner are shown in Fig. 4b. The HUs measured for the six samples labelled with their density to the immediate right, in the Fig. 4a, were higher for the GE image compared to the Siemens image by an average 3.7% (ratio of the difference to the mean HU).

**Fig. 5:**
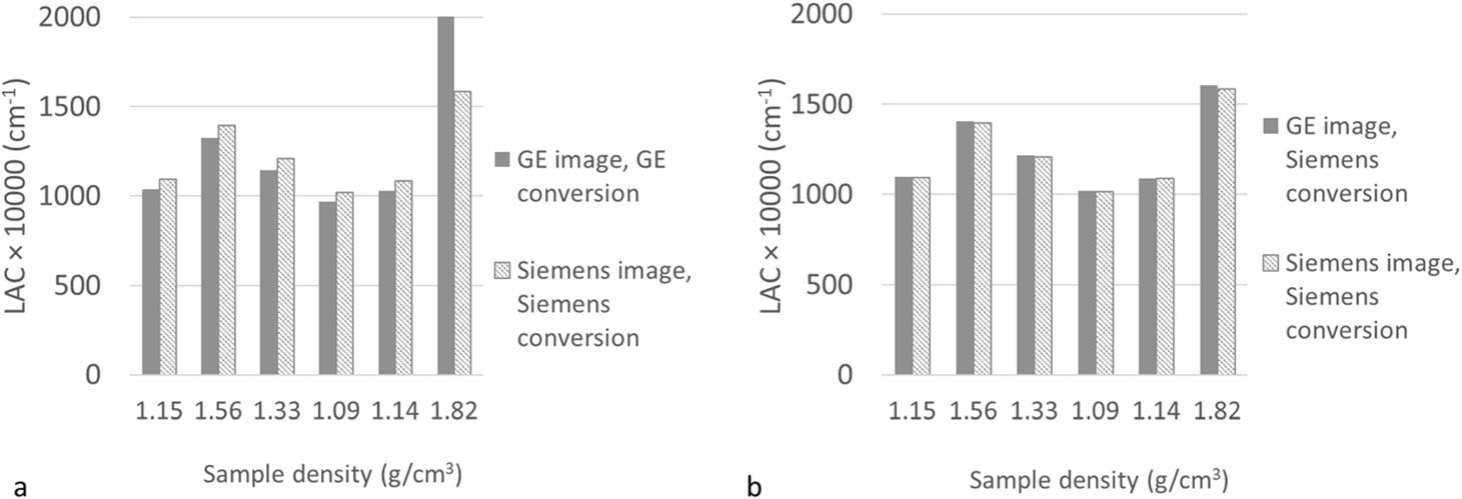
**a** LAC at 511 keV calculated from HU using the vendor specific curve. **b** LAC derived using Siemens scaling

The LACs obtained using the curves supplied by each vendor for the same six samples are given in Fig. 5a. When using the vendor’s own scaling scheme, Siemens values were greater than GE by an average of 5.3% (ratio of the difference to the mean LAC) for the five least dense samples. For the most dense sample, i.e. bone tissue density (density = 1.82 g/cm^3^, HU ~ 1200), this difference was much greater at 11.6%. The much greater difference seen at a high HU is consistent with Fig. 1 as the gradient of the Siemens curve is much greater than GE above 1000 HU.

If the HUs measured on the images acquired by the GE and Siemens CT scanners are both scaled using the Siemens conversion method (Fig. 5b), then the LACs derived from the GE CT image very closely match those from the Siemens CT image. The LAC is similar with an average difference of 0.4% for the five samples with a density ≤ 1.56 g/cm^3^ and even for the highest density material of 1.82 g/cm^3^ the difference is only 1.2%. Similar small differences were also seen when GE scaling was applied to both the Siemens and GE CT scans. It is seen that each vendor’s scaling resulted in different LACs, but the LACs obtained, using either HU to LAC scaling method, are insensitive to small (e.g. inter-vendor) differences in HU.

**Fig. 6:**
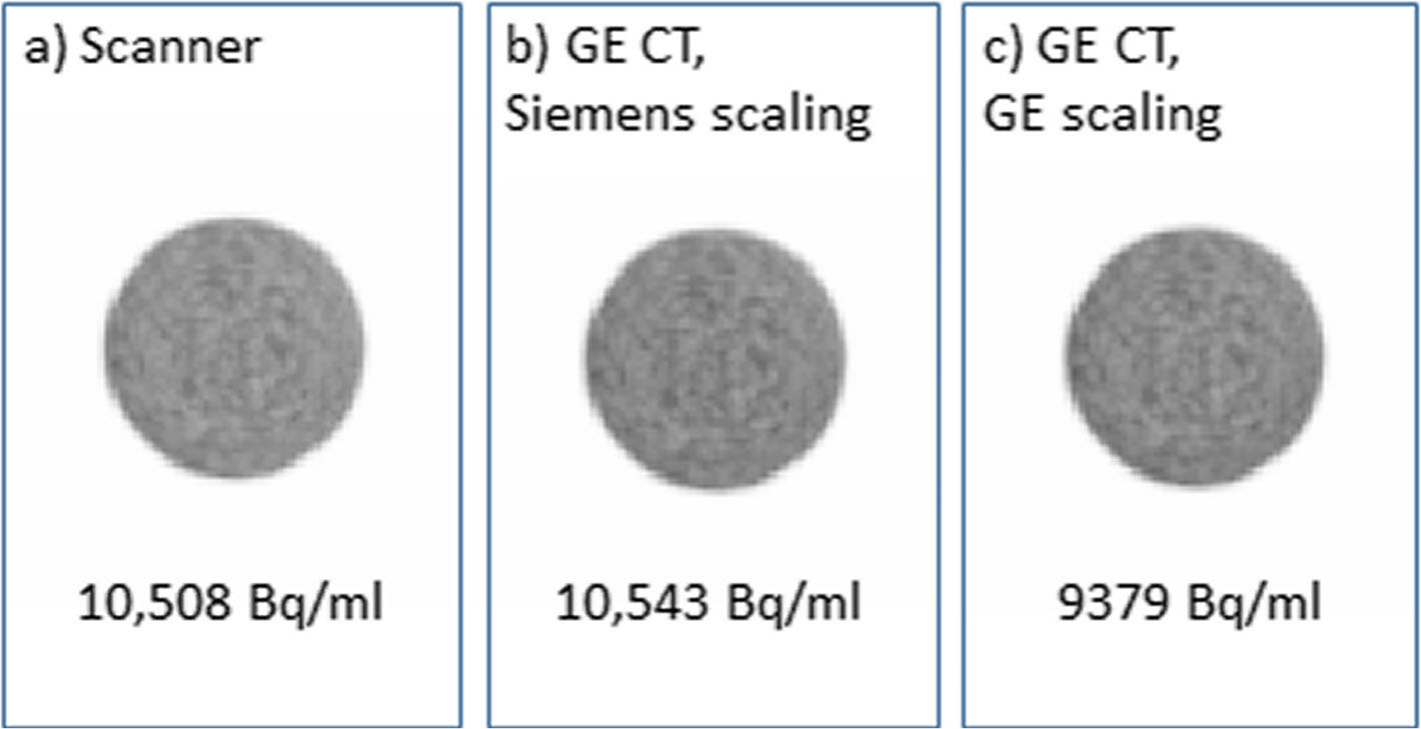
Images of the ^68^Ge daily QC phantom attenuation corrected using **a** CT provided on the scanner, **b** GE CT with Siemens HU to LAC scaling and **c** GE CT with GE HU to LAC scaling. The activity concentration measured in a large ROI located at the centre of the images is shown below each image

Figure 6 shows the ^68^Ge cylinder PET image attenuation corrected using (a) the CT-derived μ-map available on the mMR, used as the reference; (b) a GE CT scan with Siemens scaling applied; (c) a GE CT scan with GE scaling applied. The mean activity concentration measured in a large ROI located over the centre of the phantom is given below each image. For the GE CT scan with GE conversion there is a 10% decrease compared to the activity concentration measured when using the built-in Siemens CT scan. This is consistent with Fig. 5a, as the GE estimated LACs are lower than Siemens so the attenuation correction factors will be too small resulting in a PET image with lower apparent activity concentration. Applying Siemens energy conversion to the GE CT image greatly reduces the difference in activity concentration to less than 0.5% measured in the reconstructed PET image. Therefore, provided the scaling curve for attenuation correction of a PET scan matches that used in the reconstruction of the PET image with which the scanner is calibrated, the calibration factor remains valid. Otherwise, an adjustment of this factor would be required to account for the difference in estimated LAC.

### Effect of pads used to position and keep the patient’s head still during long brain PET-MR examinations

**Fig. 7:**
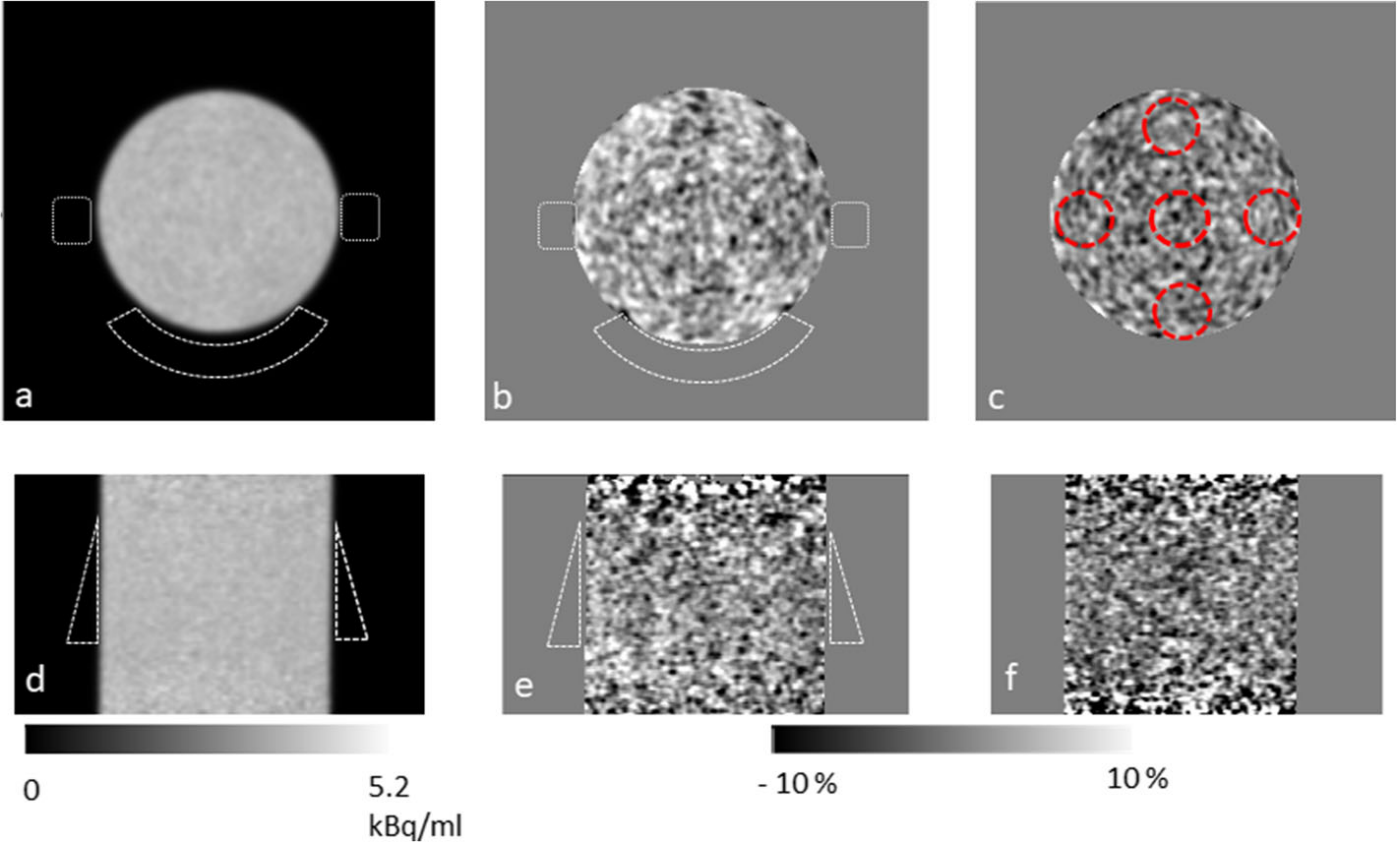
Transverse images **a**–**c**, coronal images **d**–**f**. **a**, **d** PET image acquired with pads in place. **b**, **e** Difference between PET images with/without pads attached to phantom. **c, f** Subtraction of two PET images acquired back to back. Dotted white lines represent the position of the pads in **a**, **b**, **d** and **e**. Dotted red lines in **c** show the positions of ROIs used to make measurements given in Table 1

Figure 7 shows the PET images acquired with the pads attached to the sides of the phantom along with the percentage difference between the PET acquired with and without the pads present. Also shown is an image of the difference between two scans acquired sequentially in the absence of pads with no change in imaging condition.

The percentage difference between ROIs, positioned as shown in Fig. 7c, in the difference images given is shown in Table 1. For comparison, results are included for the small shift of approx. 2 mm imposed between the PET and the CT-derived μ-map orthogonally to the long axis of the scanner. Although the decrease between the ROI measurements made with the pads on and off is greater (average = 1.7%) than the difference between two sequentially acquired scans (average = 0.4%), it is smaller than the 3.1% decrease seen at the top of the phantom when a 2-mm drop in the position of the CT is imposed. Furthermore the percentage decrease in activity concentration caused by the pads is small compared to the standard deviation of the difference.

**Table 1:** Average ROI values taken from an image of the percentage difference between two PET scans. The standard deviation is quoted in brackets for each measurement

ROI position	Diameter × length ROI (mm)	% difference pads on vs pads off	% difference between two sequential scans	% difference with 2 mm downward shift between the PET and CT
Anterior	50 × 60	1.24 (3.97)	0.42 (4.03)	3.05 (7.3)
Posterior	50 × 60	2.29 (3.74)	0.34 (4.01)	− 0.01 (5.4)
Left	50 × 60	1.75 (4.03)	0.48 (3.86)	1.31 (5.8)
Right	50 × 60	1.33 (4.19)	0.34 (4.01)	1.32 (5.7)
Centre	100 × 60	1.8 (4.2)	0.35 (3.36)	1.98 (6.05)

### Effect of dense hair in the FOV

**Fig. 8:**
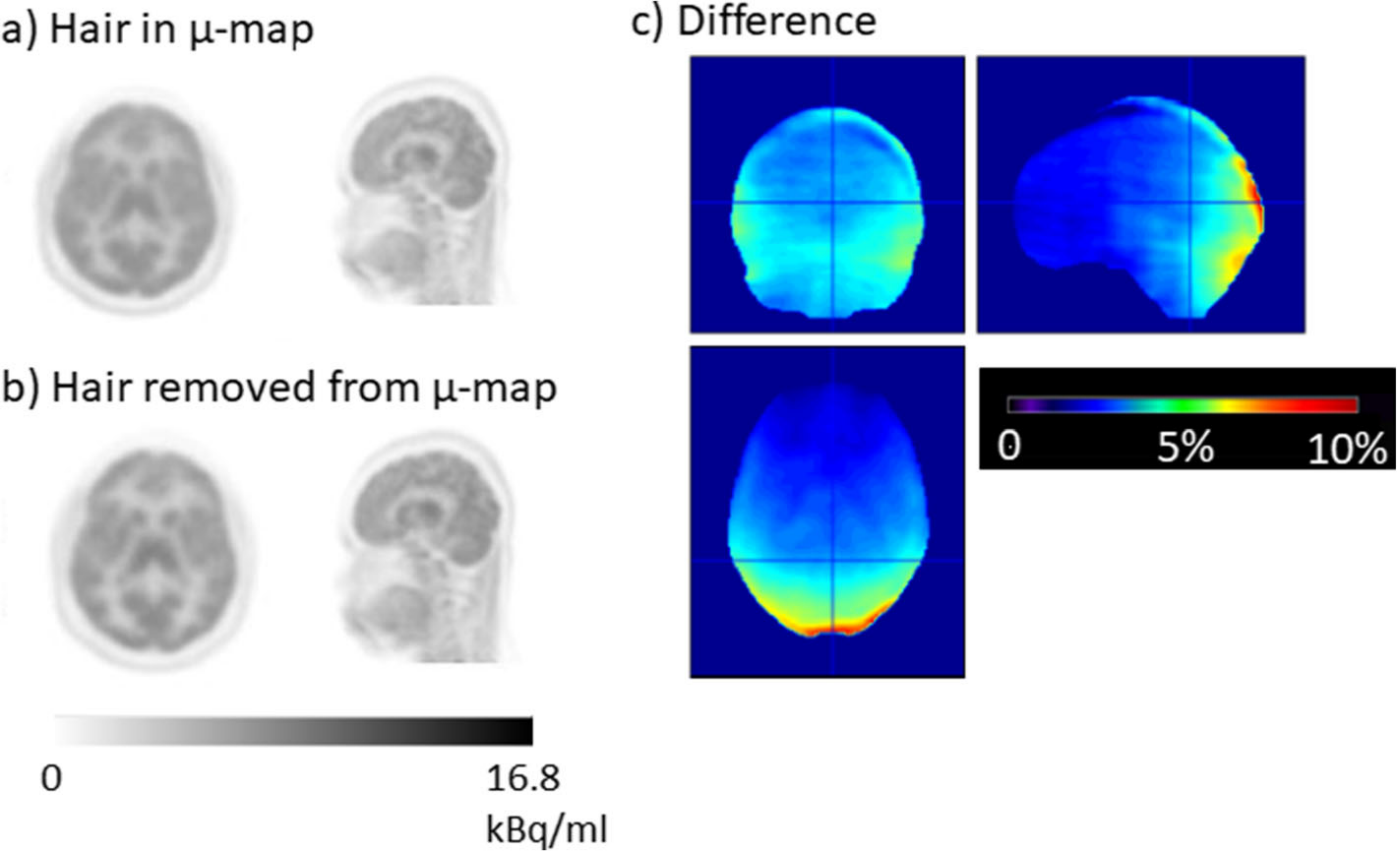
**a** PET scan reconstructed with the hair in the μ-map. **b** PET scan reconstructed with the hair removed from the μ-map. **c** Transverse, coronal and sagittal view showing the percentage difference between the PET image attenuation-corrected using a CT with the hair included compared to the image corrected using a CT with the hair masked out

A percentage difference image between a PET image reconstructed using a CT scan that included the participant’s dense hair (Fig. 2(d-f)) and one in which most of the dense hair had been manually masked out is shown in Fig. 8c alongside the two PET images reconstructed with (Fig. 8a) and without (Fig. 8b) the hair present in the μ-map. The difference in Fig. 8c is large at >10 % towards the back of the head, next to the location of the hair. This implies that the effect of dense hair, when not included in the μ-map, can be significant.

**Fig. 9:**
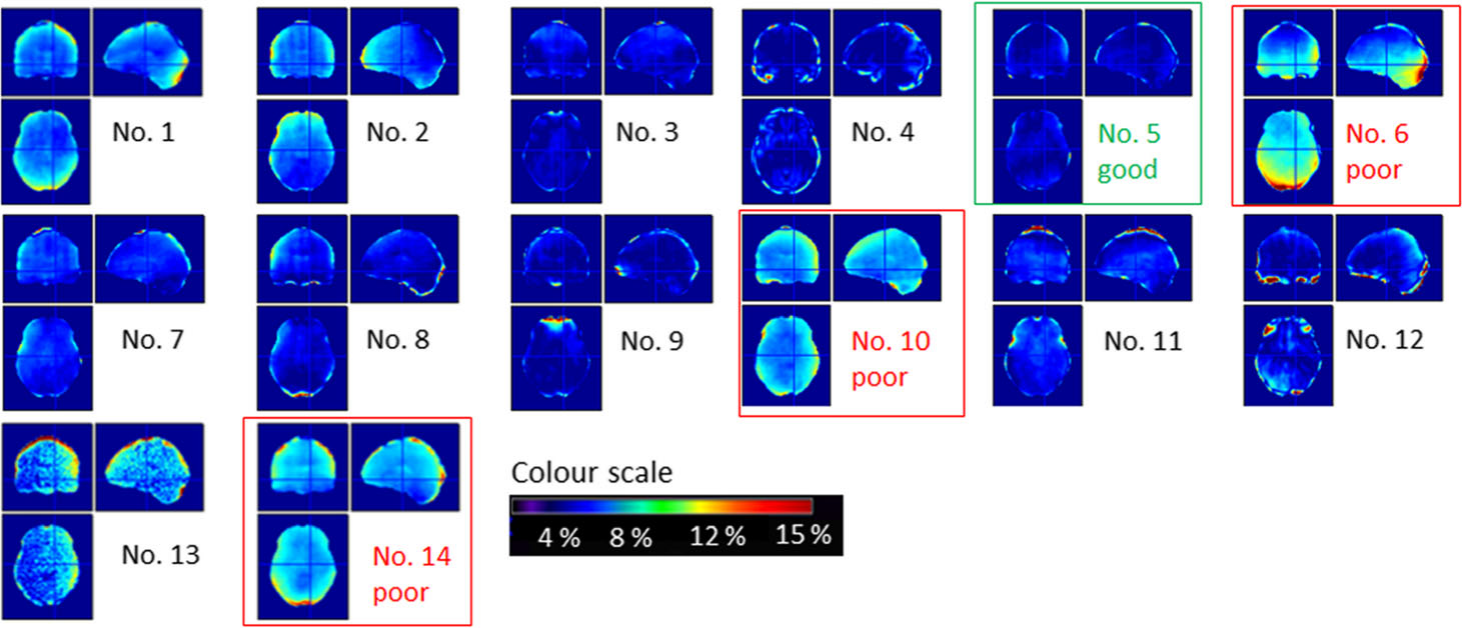
Percentage differences between PET images attenuation-corrected using a CT scan (includes hair) and a pseudo CT from the MaxProb method (doesn’t account for hair).

Figure 9 shows the percentage difference between PET images that were attenuation corrected using the MaxProb method (which does not account for hair) and PET images attenuation-corrected using CT (includes hair). Dense hair surrounds the patient’s head in the three worst performing cases outlined in red (labelled 6, 10 and 14). In the case highlighted in green (labelled 5), in which only small errors can be seen, very little hair appeared around the head. Although results were confounded by other issues (e.g. μ-map to PET registration accuracy), hair (with HU <− 300) was seen in the CT images for all poorly performing cases, as well as in the highlighted extreme examples. Furthermore, the location of the discrepancy appears to follow the shape of the hair, e.g. in no. 1 the patient was wearing their hair in a ponytail at the back of the head and in no. 13 clearly defined braids were responsible for the mottled pattern seen in the difference image. This would imply that not accounting for the hair can affect the accuracy of attenuation correction in the brain and is therefore a limitation of atlas-based methods such as MaxProb and any other method that does not take account of the patient’s hair.

### Effect of headphones and safety considerations when they are not worn

**Fig. 10:**
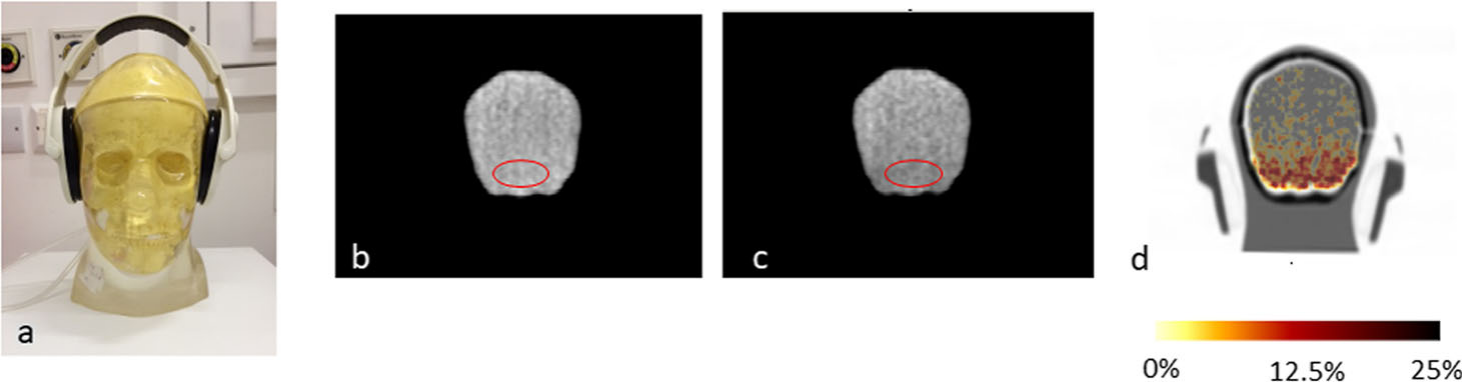
**a** Photograph of head phantom with headphones in place **b** PET image acquired without the headphones on, **c** PET image acquired with headphones positioned as in **a**, **d** the difference between the image with and without the headphones, as a percentage of the image without the headphones, superimposed onto a CT scan of the phantom with the headphones covering the ears

The PET image of the brain phantom with and without the headphones present, alongside an image of the difference, is shown in Fig. 10. For reference a photograph of the head phones on the phantom is also shown. A large average 13% decrease in activity concentration was calculated for the uniform region between the headphones at the base of the brain compartment shown in Fig. 10b, c. This error implies that, if they cannot be incorporated into the μ-map, headphones should not be worn for brain imaging.

The *L*_Aeq_ and *L*_Zpk_ measured for four example brain research protocols are given in Table 2. The table includes the noise levels experienced by the patient assuming the patient is wearing ear plugs. The *L*_Aeq_ and *L*_Zpk_ experienced by the participant for all four studies are below the exposure limits of 85 dB(A) and 140 dB respectively given in the European Community standard provided in 60601-2-33 [26]. This implies that scanning can be safely performed with ear plugs alone.

**Table 2:** *L*_Aeq_ and *L*_Zpk_ measured for four different brain research protocols. The noise levels experienced by the participant (columns 3 and 5) take into account 12.5 dB noise reduction by the ear plugs

Study number	Measured *L*_Aeq_, dB(A)	*L*_Aeq_ experienced by participant, dB(A)	Measured *L*_Zpk_, dB	*L*_Zpk_ experienced by the participant, dB
1	93.5	81.0	110.5	98.0
2	92.8	80.3	112.5	100.0
3	94.3	81.8	126.6	114.1
4	91.9	79.4	144.5	132.0

## Discussion

Although Siemens and GE use different curves to convert HU to LAC, provided the scaling applied is the one used by the manufacturer of the PET-MR, it is possible to use a CT scan acquired on a PET-CT system of the other vendor and still be able to accurately quantify PET uptake in a reconstructed PET image. The LAC remains approximately the same for a range of density materials for a CT scan acquired on either a Siemens or GE PET-CT scanner provided the same scaling is used for both CT scans. The reason that the difference, seen in HU between Siemens and GE, is masked, once the scaling to 511 keV has been applied, is due to the low gradient of the scaling curves. Therefore a small difference in HU leads to much smaller percentage difference in LAC. Although we have only demonstrated this for a tube voltage of 120 kVp, all scaling curves from 80 kVp to 140 kVp have a similar form, which will mask any difference seen in the HUs between the two scanner types when converted to LAC.

We have further demonstrated on the mMR that only a small difference of 0.3% in measured activity concentration in a uniform cylinder results when a GE CT scan is used for attenuation correction with Siemens scaling applied. This suggests that although there is a difference between HUs and LACs scaled using the CT vendor’s own curves, provided the curves used by the vendor of the PET-MR scanner are applied, the CT-derived μ-maps from either scanner are very similar and can be used to generate PET images with the same activity concentration. The accuracy of this correction will be approximately the same as the accuracy of attenuation correction on the PET-CT made by the vendor of the PET-MR system.

The pads used to keep the patient’s head still in the mMR head coil reduce the PET signal by only a small amount. As shown in Table 1, a difference of 1.8% is observed due to the presence of the pads which is greater than the difference of 0.3 % seen between two PET images acquired sequentially in identical circumstances. Having the pads in place in the head coil is advisable to avoid patient motion. It would also be impractical to use a fiducial marker-based method to add the pads into the μ-map for all the images in a brain research study. From Table 1, a small downward shift of 2 mm (the approximate magnitude of the type of small shift seen due to patient motion) between the PET image and the μ-map has resulted in a 1.98% error in a large central ROI, with an error of 3.05% seen at the top the phantom. This type of heterogeneous error could have a greater effect on results, than the small global error caused by the pads, as brain research studies often rely on regional comparisons. Furthermore, the experiment we described here was more sensitive to the effect of the pads than in a patient study when the pads would not be directly in contact with the activity of the brain. It can be concluded that the pads are having only a minimal effect and that not using them could make analysis of brain scans more prone to error caused by motion.

The presence of the dense hair, shown in Fig. 2d–f, does appear to cause a large error of > 10% at the back of the patient’s head if the hair is not included in the μ-map (Fig. 8). The poor performance of the atlas-based algorithm in the highlighted cases in Fig. 9 has been attributed to the hair not being included in the atlas-based μ-map, although the algorithm method appears to perform well in at least half of the cases, which is consistent with [6, 10]. When compared to the subjects recruited for these two studies, we may have seen more subjects with densely woven hair (e.g. braids or dreadlocks) in our evaluation of MaxProb, due to the local population recruited in London.

Not accounting for the presence of dense hair is not only an issue for MaxProb and other atlas-based methods that do not incorporate hair in the CT-derived μ-map but also for almost all methods used routinely for PET-MR attenuation correction. The hair is unlikely to be accounted for on a segmented MR image and therefore not included in the attenuation map. This applies to the Dixon, UTE and ZTE methods available on commercial systems. To improve on the potential errors imposed through attenuation by the hair, the subject could be asked to wear their hair down and spread it out as much as possible. If a hairstyle is particularly elaborate, either the subject could be excluded from the study, or a very carefully drawn ROI could be used to segment out the bed whilst the hair remains in a CT-derived μ-map for accurate attenuation correction.

In clinical FDG brain reporting, visual interpretation is used to assess for significant hypo-metabolism. If a patient were to be scanned without taking any of the mitigating steps to reduce the decrease in apparent uptake due to the presence of hair, it could affect interpretation of a patient’s scan for dementia or epilepsy. As the presence of hypo-metabolism in occipital regions is used to differentiate Alzheimer’s dementia and Lewy body dementia, the artificial decrease in uptake shown in the worst performing case (no. 6 in Fig. 9) could potentially confound the ability of the clinician to interpret the PET image correctly. Also in patients with epilepsy, artefactual decreases in uptake could interfere erroneously with genuine reduced activity caused by a hypo-metabolic focus. Anatomical MR images, acquired at conventional echo times, would not necessarily indicate the presence of hair and therefore the clinician may be unaware of potential problems when reporting a PET-MR study. For PET-CT reporting, the CT is often viewed to check for anything unusual about the CT scan that could affect PET attenuation correction.

The use of headphones should be avoided when acquiring PET data on a PET-MR scanner. The phantom results reported above are consistent with previous published works [17–20]. In previous work by Ferguson et al. [17], the headphones were added into the attenuation map, using fiducial markers located on them and image registration. As the headphones would need to be added to the μ-map in a varying position for every subject in a research study it would be difficult to use this technique in practice. Furthermore, even with this correction there were still errors recorded of over 4%.

If ear plugs are worn without the additional protection provided by headphones, depending on local institutional policies, a sound risk assessment may need to be performed in order to ensure patient safety as part of the workup for starting a new research study. For the four example research studies presented above, the sound levels recorded were below the limits given in [26], implying that it is safe to scan in the absence of headphones. If communication with the subject is important the use of low attenuating headphones could be considered [19]. However previous work has reported that communications only improved slightly when using headphones during PET-MR brain scans [18], suggesting that the need of headphones during brain scans could be eliminated altogether.

## Conclusion

There are a number of issues that affect the accuracy of attenuation correction in brain PET-MR imaging. However, for most of the problems, there are simple, pragmatic solutions to minimise each issue, e.g. removal of headphones for brain imaging to avoid the complex registration problem of including them in the attenuation map. Furthermore, some of the bias reported in this work when dense hair or headphones are present in the field of view and not accounted for in the attenuation maps, could potentially be reduced, although not necessarily removed, on scanners with TOF capability (e.g. the GE SIGNA) since TOF PET reconstruction is less sensitive to inaccuracies in the attenuation map used during reconstruction [27, 28]. Nevertheless, the practical limitations of attenuation correction in PET imaging on a PET-MR scanner, such as those highlighted here, may only be fully and robustly solved using a different approach to attenuation correction, e.g. the use of a transmission source in the scanner such as that proposed by Renner et al. [29] or emission-derived attenuation correction image reconstruction [30, 31].

Although we describe here our experience of brain attenuation correction on a PET-MR scanner, there will be additional potential issues that could cause inaccuracies such as the positioning of the coil in the μ-map, the effect of any other objects in the PET FOV, e.g. the small detachable mirror often located on the head coil etc. There are also practical challenges to routinely processing PET data with non-vendor provided or non-vendor approved methods even if they provide an accurate method of AC. For example if reverting to CT for PET-MR attenuation correction purposes, these will include the logistics of scheduling an additional CT scan, justification of the associated radiation dose, though relatively small compared to that from the radiotracer, and developing a user friendly data processing pipeline.

Many of the limitations/inaccuracies described here for the brain will also apply to attenuation correction of other regions of the body. However, these could be minor compared to other confounding issues associated with imaging areas outside of the brain, e.g. cardiac and respiratory motion, the difficulty of image registration if CT or atlas-based methods are considered, and the phased array surface coils not being included in the μ-map. As such, the practical evaluation of body attenuation remains the topic of future investigation. Furthermore, body attenuation correction is likely to be application/region specific rather than a generic whole-body solution.

## Data Availability

According to the Wellcome Policy on data, software and materials management and sharing, all data supporting this study will be openly available at https://kclpure.kcl.ac.uk/portal/files/126769902/Manuscript_jmackewn_trackchanges_comments2_pureupload.pdf.
