# Correction to: Practical issues and limitations of brain attenuation correction on a simultaneous PET-MR scanner

**DOI:** 10.1186/s40658-020-00312-z

**Published:** 2020-07-14

**Authors:** J. E. Mackewn, J. Stirling, S. Jeljeli, S-M Gould, R. I. Johnstone, I. Merida, L. C. Pike, C. J. McGinnity, K. Beck, O. Howes, A. Hammers, P. K. Marsden

**Affiliations:** 1 King’s College London and Guy’s and St Thomas’ PET Centre, School of Biomedical Engineering and Imaging Sciences, King’s College London, London, UK; 2 Guy’s and St. Thomas’ NHS Foundation Trust, London, UK; 3 ERMEP-Imagerie du vivant, Lyon, France; 4 Department of Psychosis Studies, Institute of Psychiatry, Psychology and Neuroscience, King’s College London, De Crespigny Park, London, UK; 5 South London and the Maudsley NHS Foundation Trust, London, UK; 6 MRC London Institute of Medical Sciences, Hammersmith Hospital Campus, London, UK

**Correction to: EJNMMI Phys (2020) 7:24**

**https://doi.org/10.1186/s40658-020-00295-x**

Following publication of the original article [1], the authors identified an error in the author name of C. J. McGinnity and S-M Gould.

The incorrect author names are: C. G. McGinnity and S. M. Gould

The correct author names are: C. J. McGinnity and S-M Gould

The author group has been updated above and the original article [1] has been corrected.
